# MiR‐363‐5p modulates regulatory T cells through STAT4‐HSPB1‐Notch1 axis and is associated with the immunological abnormality in Graves' disease

**DOI:** 10.1111/jcmm.16876

**Published:** 2021-08-25

**Authors:** Xianlun Yin, Junfeng Ge, Xiurong Ge, Jing Gao, Xinhuan Su, Xiaowei Wang, Qunye Zhang, Zhe Wang

**Affiliations:** ^1^ The Key Laboratory of Cardiovascular Remodeling and Function Research Chinese Ministry of Education Chinese National Health Commission and Chinese Academy of Medical Sciences The State and Shandong Province Joint Key Laboratory of Translational Cardiovascular Medicine Department of Cardiology Qilu Hospital Cheeloo College of Medicine Shandong University Jinan China; ^2^ Department of Anesthesiology Jinan Second People's Hospital Jinan Shandong China; ^3^ Division of Endocrinology and Metabolism Division of Geriatrics Shandong Provincial Hospital Cheeloo College of Medicine Shandong Provincial Key Laboratory of Endocrinology and Lipid Metabolism Shandong Institute of Endocrine and Metabolic Disease Shandong University Jinan China

**Keywords:** autoimmune disease, Graves' disease (GD), miR‐363‐5p, regulatory T cells (Tregs), STAT4

## Abstract

MiRNAs are a class of small non‐coding RNAs with ability to regulate function of Treg cells and are involved in many autoimmune diseases. Our previous study found that miR‐363‐5p expression was significantly upregulated in peripheral Treg cells of GD patients. Herein, we aimed to investigate its effect and mechanism on Treg cell dysfunction in GD patients. The results showed that miR‐363‐5p upregulation was significantly associated with the Treg cell dysfunction and inflammatory factors levels in GD patients. Transcriptome sequencing revealed that 883 genes were significantly regulated by miR‐363‐5p in Treg cells. These genes with significant differential expression were primarily involved in lymphocyte differentiation, immunity, as well as Notch1 and various interleukin signalling pathways. Moreover, miR‐363‐5p can regulate HSPB1 and Notch1 through the target gene STAT4, thereby regulating Notch1 signalling pathway and inhibiting Treg cells. The effects of miR‐363‐5p on Treg cell function and STAT4‐HSPB1‐Notch1 axis were also verified in GD patients. In conclusion, our results indicated that miR‐363 could inhibit the proliferation, differentiation and function of Treg cells by regulating the STAT4‐HSPB1‐Notch1 axis through target gene STAT4. MiR‐363‐5p may play an important role in Treg cell dysfunction and immune tolerance abnormalities in GD patients.

## 1 INTRODUCTION

Graves' disease (GD) is a common organ‐specific autoimmune disease. The most prominent pathological features of GD include thyroid‐specific autoantibody, lymphocytic infiltration and diffuse hyperplasia of thyroid tissue.^1^ The pathogenesis of GD is very intricate. The long‐term complex interactions of many genetic (HLA‐DR, CTLA‐4 and PTPN22, etc.) and environmental factors disturb immune homeostasis, which destroy the immune tolerance of thyroid tissue and result in the production of autoantibodies. Among these autoantibodies, the thyroid‐stimulating antibody (TSAb) plays critical roles in GD development.^2^ However, the exact mechanisms involved in the breakdown of immune tolerance to TSHR and TSAb production have not been fully elucidated until now.

Regulatory T cell (Treg), an important CD4+ T‐cell subset, is critical for immune regulation and plays a central role in the maintenance of autoimmune tolerance through secretion of inhibitory cytokines (e.g. IL‐10 and TGF‐β) and cell‐to‐cell contact inhibition.^3^ Mutations in FOXP3, a key transcription factor for Treg cell development and function, could cause severe dysfunction of Treg cells and result in IPEX syndrome, a serious multisystem autoimmune disease. In several autoimmune diseases such as psoriasis and systemic lupus erythematosus (SLE), there are significant abnormalities in the number or function of Treg cells.^4^, ^5^ GD is a common autoimmune disease. Many studies have shown that the quantity and/or function of Tregs are decreased in GD patients.^6^, ^7^ Nonetheless, most of the current research on Tregs in GD patients focused on the description of abnormalities, while the molecular mechanisms behind these anomalies are still not fully investigated. The reported mechanism studies have also focused on key functional proteins of Treg cells such as FOXP3 and IL‐10.^8^, ^9^ However, Treg functions are regulated by many molecules, including not only proteins such as cytokines and transcription factors, but also other molecules such as prostaglandins and nucleic acids. A thorough understanding of the functional regulation of Tregs and the mechanisms by which Tregs are abnormal in GD patients requires an in‐depth study of these non‐protein regulatory molecules.

MiRNAs are 18‐25nt non‐coding RNAs with regulatory functions. They play critical roles in the development and function of immune system. Dicer is the key RNase that regulates maturation of various miRNAs. Treg‐specific ablation of Dicer causes severe systemic autoimmune diseases in mice, similar to that in mice whose Tregs are eliminated directly, suggesting that miRNAs are key regulators of development, proliferation and function of Tregs.^10^ Several miRNAs had been reported to play vital roles in Treg development and T cell‐dependent antibody responses by targeting Foxp3.^11^ MiR‐106b could regulate Treg differentiation and maturation by targeting CDKN1A/p21 to regulate TGF‐β pathway.^12^ MiR‐17 could also regulate Foxp3 expression and function.^13^ However, there are also conflicting results on the roles of miRNA in Treg function. Some studies reported that miR‐21 negatively regulated Foxp3 expression and Treg ratios in adult peripheral blood mononuclear cells (PBMCs), but other study showed that Foxp3 expression was positively regulated by miR‐21.^14^, ^15^ This implies that the roles and mechanisms of miRNAs in Tregs function are complex and more intensive efforts are needed to elucidate them.

Accumulating evidences demonstrated that miRNAs play key roles in the development various autoimmune diseases, such as systemic lupus erythematosus and multiple sclerosis. MiR‐409‐3p and miR‐1896 could synergistically exacerbate experimental autoimmune encephalomyelitis in mice.^16^ The serum levels of several miRNAs in GD patients were also found to be significantly abnormal.^17^ Our previous studies showed that the expression of some miRNAs was significantly changed in peripheral Tregs of GD patients, causing abnormalities in multiple signalling pathways.^1^ Some of them have been confirmed in other studies.^17^ However, whether these miRNAs with significantly changed expression in Treg cells are involved in the Treg dysfunctions, what roles they play in the abnormalities of Tregs in GD patients, and what molecular mechanisms are involved have not yet been investigated.

Herein, we found that the number and function of Tregs were significantly decreased compared with healthy individuals. Meanwhile, miR‐363‐5p expression in Tregs was significantly upregulated, which was closely associated with immune inflammation and thyroid dysfunction in GD patients. MiR‐363‐5p could inhibit proliferation/differentiation and function of Tregs by regulating STAT4‐HSPB1‐Notch1 axis through its target gene STAT4. This might be one of the important mechanisms for the abnormalities in Tregs in GD patients. Our findings provided valuable clues to reveal the mechanisms of immune abnormalities in GD patients.

## 2 MATERIALS AND METHODS

### 2.1 Ethics statement

This study has been approved by the Ethics Committee of Shandong Provincial Hospital (NO 2015–054) and conformed to the Declaration of Helsinki. All subjects were informed of the nature of the study and were provided informed consent. All procedures were performed in compliance with relevant laws and institutional guidelines.

### 2.2 Recruitment of subjects and sample collection

Twenty GD patients and healthy individuals were recruited from Shandong Provincial Hospital. GD patients were diagnosed according to the medical history, physical examination and laboratory tests including TSH, free triiodothyronine (FT3), free thyroxine (FT4) and TRAb based on 2018 European Thyroid Association Guideline for the Management of Graves’ Hyperthyroidism.^18^ The exclusion criteria were as follows: smoking, pregnancy, alcohol addiction, hypertension, diabetes mellitus, lipid dysregulation, BMI>27; usage of hormonal medication; and medical history of malignancy. Peripheral blood (5 ml) was collected from all subjects in the morning after an overnight fast (≥8 h). The serum samples were stored in −80°C for subsequent experiments.

### 2.3 Flow cytometric analysis and Cytokine assay

Peripheral blood mononuclear cells were purified by Ficoll density centrifugation (Sigma‐Aldrich) and stained with anti‐CD4‐FITC (BD Bioscience), anti‐CD25‐APC (eBioscience) for 30 min at 4°C in the dark. Following fixation and permeabilization, cells were stained with anti‐Foxp3‐PE (eBioscience). Flow cytometric analysis was performed using FACSCalibur (Becton Dickinson), and the data were analysed by FlowJo X software (Flow Jo). The levels of cytokine IL‐2, sCD25, IL‐10 and sCD14 were detected by the Multiplex Human Premixed Multi‐Analyte Kit (R&D, USA) and high sensitivity ELISA kits (eBioscience), respectively. All procedures were performed according to the user manual.

### 2.4 Cell culture and transfection

HEK293T and CEM/C1 cells purchased from Shanghai Cell Bank of Chinese Academy of Sciences were cultured in DMEM (Gibco, Thermo Fisher Scientific) containing 10% foetal bovine serum (Biological Industries) at 37°C with 5% CO_2_. CD4^+^T cells isolated from PBMC were cultured in RPMI 1640 medium containing 10% foetal bovine serum (Gibco, Thermo Fisher Scientific) at 37°C with 5% CO_2_. The miR‐363‐5p agomir, antagomir (defined as miR‐363 and anti‐miR‐363 in the Figures) and negative control sequences (Ribobio) as well as pCMV6‐HSPB1 (Origene) and pCMV3‐STAT4 expression vectors (Sino Biological), respectively, were transfected into cells using Lipofectamine RNAiMAX or Lipofectamine 3000 reagent (Life Technologies). Then, samples from each group were collected for subsequent experiments.

### 2.5 RNA extraction and Real‐time quantitative PCR

Total RNA was extracted using TRIZOL reagent. MiRNA expression was detected by real‐time PCR, and locked nucleic acid primers were purchased from TaKaRa. Human RNU6B was used as an internal reference. The mRNA expressions of STAT4, HSPB1, Notch1, IL‐10 and Foxp3 were analysed using SYBR Green qPCR, and their primers were synthesized by Sangon Biotech. Human ACTB was used as an internal reference. Reverse transcription kit and TB Green Premix Ex Taq kit were purchased from TaKaRa. All primers used in this study are presented in Table S1.

### 2.6 Transcriptome sequencing and data analysis

The mRNA was purified from 5 μg total RNA using poly‐T oligo‐attached magnetic beads. After ultrasonic fragmentation, sequencing libraries were prepared using TruSeq RNA Library Prep Kit (Ilumina). PE150 sequencing was then performed on an Illumina HiSeq 2500 (Illumina). After removal of low‐quality reads and sequences of adaptors and primers, all clean data were aligned with UCSC human reference genome (hg19) using HISAT package. The expression levels of all detected genes were standardized (FPKM, Fragments per Kilobase Million) using StringTie and ballgown. The genes with significantly greater than 1.5‐fold change in expression levels were considered as differential genes. Pathway and functional enrichment analysis of target genes as well as differentially expressed genes was carried out using Enrichr online tools.^19^, ^20^

### 2.7 Western Blotting

Cells were lysed by RIPA lysis buffer containing 1 mmol/L PMSF (Beyotime). After centrifugation at 12,000 *g* for 15 min, the supernatant was collected and protein concentration was determined using BCA (Thermo). Total protein was electrophoresed on 10% SDS–PAGE and electrically transferred to PVDF membranes (Bio‐Rad). After blocking, the membranes were incubated with anti‐β‐actin (Santa Cruz Biotechnology), anti‐phospho‐Stat4 (Tyr693) and anti‐Notch1 mAb (Cell Signaling Technology) overnight at 4°C. After washing, the membranes were incubated with the peroxidase‐conjugated secondary antibody (Proteintech Group) for 2 h. Protein bands were detected using ECL kit. Densitometry analysis was performed by Image pro plus 6.0 software (Media Cybernetics).

### 2.8 Dual‐luciferase reporter assay

The human STAT4 3'UTR region containing the predicted binding sequence of miR‐363‐5p seed sequence was amplified with normal primers (WT), and the mutant sequence of the aforementioned 3'UTR region was amplified with mutation‐site primers (Mut). Then, these sequences were cloned into pmir‐GLO vector (Promega) to construct wild‐type and mutant luciferase reporter vectors, respectively. Next, these reporter vectors were co‐transfected into 293T cells with miR‐363‐5p or corresponding negative control sequence (Ribobio), respectively. After transfection for 36 h, the luciferase activity was measured using Dual‐Luciferase Reporter Assay System (Promega).

### 2.9 Assay for proliferation and immunosuppressive function of Treg

Peripheral blood mononuclear cells of healthy individuals were isolated by Histopaque‐1077 (Sigma). Then, CD4^+^T cells were purified by human CD4 MicroBeads kit (Miltenyi Biotec) and cultured in RPMI 1640 medium containing 10% foetal bovine serum (Gibco, Thermo Fisher Scientific). Next, CD4+ T cells were transfected with different oligonucleotides and were stimulated to differentiate by CD3/CD28 (Gibco, 11131D, cell:bead = 4:1) and IL‐2 (Gibco, PHC0021, 100 U/ml) as described in previous studies.^21^, ^22^, ^23^ Five days later, the proportion of Treg cells was measured by flow cytometry. Treg cells were sorted from human PBMCs using a FACSAria II flow cytometer (Becton Dickinson). CD8+ effector T cells sorted by flow cytometer were labelled with 2 μmol/L carboxyfluorescein succinimidyl ester (CFSE) and then were cocultured with Tregs at graded concentrations (Treg:T_effect_^ ^= 1:0.5, 1:1, 1:4). After 5 days of stimulation culture, the inhibition of effector T cells by Treg cells was analysed using flow cytometry. The suppression percentage is determined according to the following formula: suppression % = 100 × (% proliferation Teff alone−% proliferation Treg:Teff)/% proliferation Teff alone. The purity of all sorted cells was at least 95%.

### 2.10 Statistical analysis

Data were presented as mean ± SEM or median (IQR). The *t* test was used to detect the significances between two groups. One‐way anova test with appropriate correction for multiple comparisons was used to detect significances between multiple groups. The *p* < 0.05 or corrected *p* < 0.05 was considered as statistically significant. All experiments were repeated independently at least three times.

## 3 RESULTS

### 3.1 Significant abnormalities in peripheral inflammatory cytokines and Treg cells of GD patients

**FIGURE 1:**
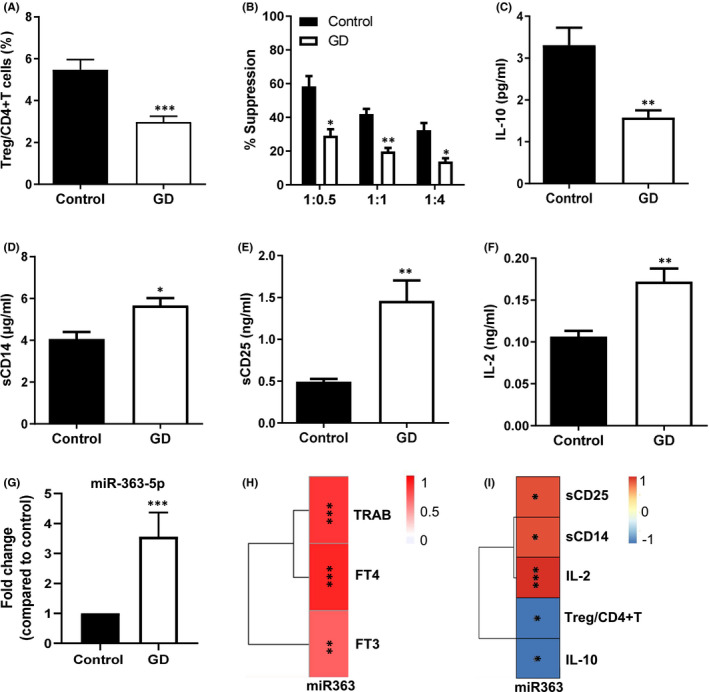
Levels of cytokine, Treg cells and miR‐363‐5p in peripheral blood of GD patients and the correlation between them and GD clinical indicators. (A) The proportion of Treg cells (CD4+CD25+Foxp3+) in the peripheral blood of healthy individuals (Control, *n* = 20) and patients with Graves’ disease (GD, *n* = 20). (B) Suppressive effect of Treg cells on effector T cells in GD patients (GD) and healthy individuals (Control). Horizontal coordinate: ratio of Treg to effector T cells. (C‐F) The serum levels of the anti‐inflammatory cytokine IL‐10 (C) and the pro‐inflammatory cytokines sCD14 (D), sCD25 (E) and IL‐2 (F) in healthy individuals (Control) and GD patients (GD). (G) Fold change of miR‐363‐5p expression in peripheral Treg cells of GD patients compared with that in healthy individuals. (H) Heatmap of the Spearman correlations between the levels of miR‐363‐5p in peripheral Tregs and TRAb, FT3 and FT4 in GD patients. (I) Heatmap of the Spearman correlations between the levels of miR‐363‐5p in peripheral Tregs and serum cytokines, Treg/CD+T cells in GD patients. Data were presented as mean ± SEM. The *t* test was used to determine statistical significance (A‐G). *: *p* < 0.05 vs. other groups; **: *p* < 0.01 vs. other groups; ***: *p* < 0.001 vs. other groups

In the present study, 20 initial patients with GD (GD group) and 20 healthy individuals (control group) were recruited. The demographic characteristics of all subjects are presented in Table 1. The levels of serum FT3, FT4 and TRAb in GD group were significantly higher than in control group (Figure S1A‐C). The percentage of Treg (CD4+CD25+Foxp3+) cells in CD4+ T cells of peripheral blood in GD patients was significantly lower than in healthy individuals (Figure 1A). The immunosuppressive function of Treg cells in the peripheral blood of GD patients was also obviously decreased (Figure 1B). Moreover, compared with healthy individuals, the serum level of anti‐inflammatory cytokine IL‐10 was markedly decreased (Figure 1C), while the serum levels of pro‐inflammatory cytokines including sCD14, sCD25 and IL‐2 were significantly increased in GD patients (Figure 1D‐F). These results suggested the significant abnormalities in immune and inflammation of patients with GD.

**TABLE 1:** Demographic characteristics of patients with Graves’ disease and healthy individuals

	Healthy individual	Graves' Disease	*p* Value
*n* (Male/Female)	20 (13/7)	20 (9/11)	ns
Age (year)	47.35 ± 9.40	41.8 ± 8.48	ns
FT3 (pmol/L)	4.73 ± 0.68	18.55 ± 9.0	***
FT4 (pmol/L)	15.68 ± 2.50	48.47 ± 27.86	***
TSH (μIU/ml)	0.0046 ± 0.0028	2.30 ± 1.28	***
TRAb (IU/L)	0.38 ± 0.16	21.44 ± 13.54	***
TPOAb (IU/ml)	924.95 ± 552.45	34.04 ± 8.01	***
TGAb (IU/ml)	12.72 ± 3.11	204.94 ± 189.39	***

Data were presented as mean ± SD.

The *t* test was used to detect significant changes.

Abbreviations: FT3, free T3; FT4, free T4; TGAb, thyroglobulin antibody; TPOAb, thyroperoxidase antibody; TRAb, thyroid‐stimulating hormone receptor antibody; TSH, thyroid‐stimulating hormone.

***p < 0.001.

### 3.2 MiR‐363‐5p was significantly upregulated in Treg cells and correlated with abnormalities in thyroid function and inflammation of GD patients

It had been reported that the expression of some miRNAs including miR‐363‐5p was significantly changed in peripheral Treg cells of GD patients.^1^ As one of the most significantly altered miRNAs, the expression changes of miR‐363‐5p in Tregs of GD patients were confirmed using real‐time PCR. The results showed that miR‐363‐5p was significantly upregulated in peripheral Tregs of GD patients compared with healthy individuals, which was consistent with previous studies reported (Figure 1G). Furthermore, Spearman's correlation analysis showed that the expression level of miR‐363‐5p in Tregs was significantly positively correlated with serum levels of several clinical indicators of GD (TRAb, FT4 and FT3) and pro‐inflammatory factors (sCD25, sCD14 and IL‐2) (Figure 1H,I, Figure S2A‐F) and was significantly negatively correlated with the ratio of peripheral Treg/CD4+ T cells and serum levels of the anti‐inflammatory factor IL‐10 (Figure 1I, Figure S2G,H). These associations indicated that miR‐363‐5p might play an important role in the immune abnormalities in GD patients.

### 3.3 The potential roles of miR‐363‐5p in immune revealed by mRNA‐seq

**FIGURE 2:**
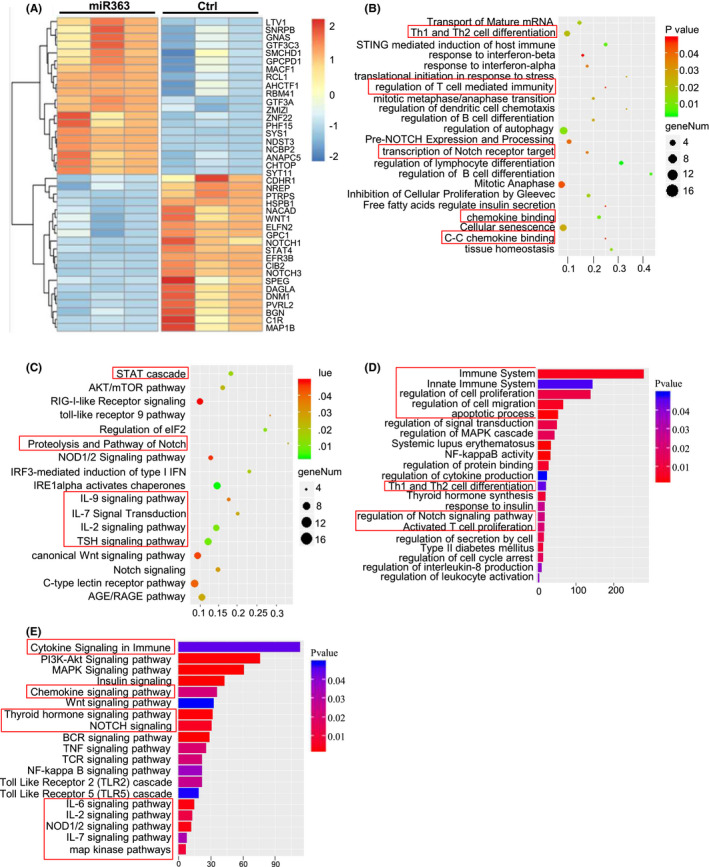
Changes in the expression profile of CD4+ T cell caused by miR‐363‐5p and potential function of miRNA‐363‐5p. (A) Heatmap of the fold changes of top 40 genes with significantly changed expressions in CEM cells transfected with miR‐363‐5p mimic (miR363) compared with CEM cells transfected with negative control sequence (Ctrl). The Wald test with Benjamini–Hochberg correction for multiple tests was used for differential expression analysis. Fold change ≥1.5 or ≤0.67 and corrected *p* ≤ 0.05 were considered significant changes. The colour bar represents log_1.5_(fold change). (B, C) Enrichment analysis of functions (B) and signalling pathways (C) of the genes significantly regulated by miR‐363‐5p in CEM cells. Colour bar represents the corrected *p* values. Bubble size represents the number of relevant genes. (D, E) Enrichment analysis of functions (D) and signalling pathways (E) of the genes in the intersection of the potential target genes predicted by multiple software and the 456 genes downregulated by miR‐363‐5p. Horizontal coordinates represent gene numbers

To reveal miR‐363‐5p function, the effects of miR‐363‐5p on the transcriptomic profiles were analysed using transcriptome sequencing in CEM cells (a CD4+T‐cell line) transfected with miR‐363‐5p agomir or negative control sequence (Ctrl). The results showed that miR‐363‐5p significantly affected on the gene expressions in CD4+ T cells. Total 427 genes were significantly upregulated, and 456 genes were downregulated (corrected *p* < 0.05; Figure S1D). The expression profiles of the top 40 genes with the most significant changes in the 883 genes regulated by miR‐363‐5p are shown in Figure 2A. The enrichment analysis indicated that miR‐363‐5p was primarily involved in several biological processes such as lymphocyte differentiation, cell cycle and transcriptional regulation, chemotaxis, lymphocyte‐mediated immunity and Th1/Th2 cell regulation, as well as the STAT, Notch1, TSH and various interleukin (IL‐2, IL‐9, IL‐7, etc.) signalling pathways (Figure 2B,C). Moreover, the target genes of miR‐363‐5p were predicted using the combination of miRDB, miRwalk, TargetScan and RNAhybrid, and the intersection of predicted target genes and the 456 genes downregulated by miR‐363 was selected. The enrichment analysis of the genes in the intersection suggested that the potential target genes of miR‐363 were mainly involved in cell migration, apoptosis, secretion and thyroid hormone synthesis and were associated with various immune diseases such as systemic lupus erythematosus and type 2 diabetes. Especially, many immune‐inflammatory functions were also significantly enriched, such as innate immunity, Th1 and Th2 cell differentiation, and T‐cell activation (Figure 2D). The pathway enrichment analysis indicated that these potential target genes of miR‐363‐5p were involved in many signalling pathways such as Notch, Nod1/2 and Toll‐like receptors, thyroid hormones, various interleukins (IL‐2, IL‐6, etc.) and cytokine/chemokine signalling pathways (Figure 2E). All these results implied that miR‐363‐5p was strongly correlated with immune response and might regulate CD4+ T‐cell function through several signalling pathways including STAT, Notch and Wnt.

### 3.4 MiR‐363‐5p targeted STAT4 to inhibit its expression

**FIGURE 3:**
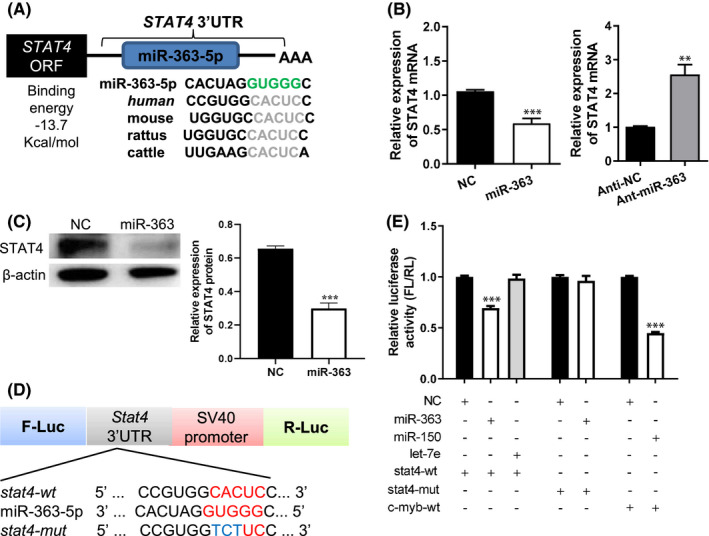
miR‐363‐5p targeted STAT4 and inhibited its expression. (A) Sequence in STAT4 3'UTR binding to the seed region of miR‐363‐5p with a low binding energy and high conservation. (B, C) Relative expressions of STAT4 at the mRNA (B) and protein (C) levels in CEM cells transfected with miR‐363‐5p agomir, miR‐363‐antagomir, agomir negative control (NC) or antagomir negative control (Anti‐NC). (D) Structural schematic diagram of the dual‐luciferase reporter vector PMI‐GLO containing the STAT4 3'UTR (STAT4‐wt) and its mutant sequence (STAT4‐mut). (E) The dual‐luciferase reporter vector containing STAT4 3'UTR (STAT4‐wt) or its mutant sequence (STAT4‐mut) and miR‐363‐5p were co‐transfected into 293T cells. Negative control sequence of miR‐363‐5p was used as control group (NC). The aforementioned two luciferase reporter vectors and let‐7e, respectively, were co‐transfected into 293T cells as a negative control. The reporter vector containing c‐myb 3'UTR, the confirmed target gene of miR‐150, was co‐transfected with miR‐150 in 293T cells as a positive control. Relative Luciferase active: fluorescence intensity ratios of firefly to sea cucumber normalized by those of 293T cells transfected with only reporter vector. Data were presented as mean ± SEM. *t* test was used to determine statistical significance in B and C. One‐way anova was used in E. **: *p* < 0.01 vs. other groups; ***: *p* < 0.001 vs. other groups

The prediction results of various software indicated that STAT4 was the target gene of miR‐363‐5p. Moreover, mRNA sequencing also suggested that miR‐363‐5p agomir could significantly suppress STAT4 expression. The 3'UTR of STAT4 in several species had a conserved binding site for the miR‐363‐5p seed sequence with a binding energy of −13.7 kcal/mol (Figure 3A). MiR‐363‐5p agomir significantly decreased STAT4 expression at both protein and mRNA levels in CEM cells. MiR‐363‐5p antagomir significantly upregulated STAT4 mRNA expression but increased STAT4 protein expression without significant differences (Figure 3B,C, Figure S3A). To clarify whether Stat4 is a target of miR‐363‐5p, a dual‐luciferase reporter plasmid containing wild‐type (STAT4‐WT) or mutant (STAT4‐Mut) sequence of STAT4 3'UTR was constructed, respectively (Figure 3D). To enhance the reliability of results, let‐7e was used as a negative control because STAT4 was not its target gene. The well‐established target gene of miR‐150, c‐myb, was used as a positive control. These plasmids were co‐transfected with miR‐363‐5p agomir in 293T cells, respectively. The results showed that the luciferase activity was significantly reduced in 293T cells co‐transfected with miR‐363‐5p agomir and STAT4‐WT plasmid, while no significant changes were observed in 293T cells co‐transfected with miR‐363‐5p agomir and STAT4‐Mut plasmid. Meanwhile, the luciferase activity was significantly decreased in 293T cells co‐transfected with miR‐150 and the plasmid containing c‐myb 3'UTR, while there was no significant change in luciferase activity of 293T cells co‐transfected with let‐7e and STAT4‐WT plasmid (Figure 3E). These results confirmed that STAT4 was the target of miR‐363‐5p.

### 3.5 MiR‐363‐5p regulated Notch1 signalling pathway through STAT4 and HSPB1

**FIGURE 4:**
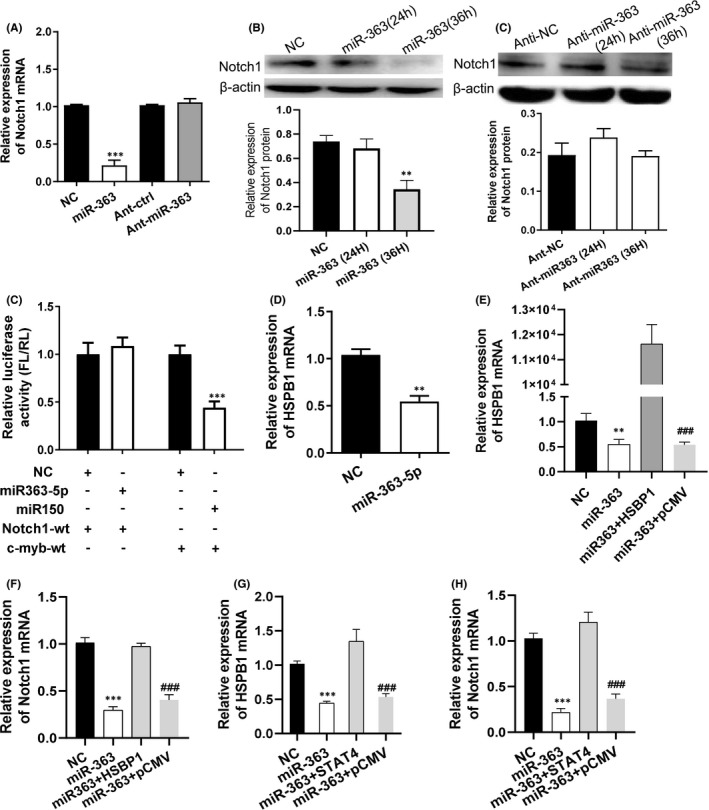
miR‐363‐5p regulated Notch1 signalling pathway through STAT4 and HSPB1. (A, B) Relative expressions of Notch1 at mRNA (A) and protein (B) level in CEM cells transfected with miR‐363‐5p agomir, antagomir or corresponding negative control sequences, respectively. NC: agomir negative control; Anti‐NC: antagomir negative control. (C) The luciferase reporter vector containing Notch1 3'UTR sequence was co‐transfected, respectively, with miR‐363‐5p or its negative control sequence (NC) into 293T cells. The reporter vector containing c‐myb 3'UTR, the confirmed target gene of miR‐150, was co‐transfected with miR‐150 in 293T cells as a positive control. Relative Luciferase active: fluorescence intensity ratios of firefly to sea cucumber normalized by those of 293T cells transfected with only reporter vector. (D) Relative expression of HSBP1 mRNA in CEM cells transfected with miR‐363‐5p or negative control sequence (NC). (E, F) Relative expressions of HSPB1 (E) and Notch1 (F) mRNA in CEM cells transfected with miR‐363‐5p, negative control sequence (NC), miR363‐5p and pCMV empty vector (miR‐363 + pCMV) or miR363‐5p and pCMV‐HSBP1 expression vector (miR‐363 + HSBP1). (G, H) Relative expressions of HSPB1 (G) and Notch1 (H) mRNA in CEM cells transfected with miR‐363‐5p, negative control sequence (NC), miR363‐5p and pCMV empty vector (miR‐363 + pCMV) or miR363‐5p and pCMV‐STAT4 expression vector (miR‐363 + STAT4). Data were presented as mean ± SEM. *t* test was performed to determine the statistical significance in (A, C and D). One‐way anova was used in (B, E‐H). In (A, C, D): **: *p* < 0.01 vs. NC groups; ***: *p* < 0.001 vs. NC groups. In B, *p* < 0.01 vs. other groups. In E‐H, **: *p* < 0.01 vs. other groups except miR‐363 + pCMV; ***: *p* < 0.001 vs. other groups except miR‐363 + pCMV; ###: *p* < 0.001 vs. other groups except miR‐363

The mRNA sequencing results indicated that, in addition to its target gene STAT4, miR‐363‐5p also regulated many other genes including Notch1, which was involved in STAT pathway. Moreover, Notch1 pathway was also significantly enriched in the signalling pathways related to miR‐363‐5p. Therefore, the relationship between miR‐363‐5p and Notch1 was analysed. The results demonstrated that miR‐363‐5p agomir significantly downregulated Notch1 expression at protein and mRNA levels in CEM cells, while miR‐363‐5p antagomir and the negative control had no significant effect on them (Figure 4A,B). The luciferase activity had no change in 293T cells co‐transfected with miR‐363‐5p agomir and the plasmid containing Notch1 3'UTR sequence. However, as a positive control, the luciferase activity was significantly decreased in 293T cells co‐transfected miR‐150 and the plasmid containing c‐myb 3'UTR sequence (Figure 4C). These results illustrated that Notch1 was not a target gene of miR‐363‐5p. Stat4, the target gene of miR‐363‐5p, is an important transcription factor regulating the expression of numerous genes, many of which modulate Notch1, such as HSPB1. Real‐time PCR showed that miR‐363‐5p significantly downregulated HSPB1 expression in CEM cells, which was consistent with mRNA sequencing data (Figure 4D). The HSPB1 expression vector could reverse the inhibitory effects of miR‐363‐5p on HSPB1 and Notch1 expression (Figure 4E,F). Also, HSPB1 was not a target gene of miR‐363‐5p (Figure S3B). Furthermore, the STAT4 expression vector could also alleviate the inhibitory effects of miR‐363‐5p on the expression of HSPB1 and Notch1 (Figure 4G,H). Thus, these results demonstrated that miR‐363‐5p could downregulate HSPB1 by suppressing its target gene STAT4, thereby repressing Notch1 expression and regulating the Notch1 signalling pathway.

### 3.6 MiR‐363‐5p suppressed Treg differentiation and function via STAT4‐HSPB1‐Notch1 axis and was associated with immune abnormalities in GD patients

**FIGURE 5:**
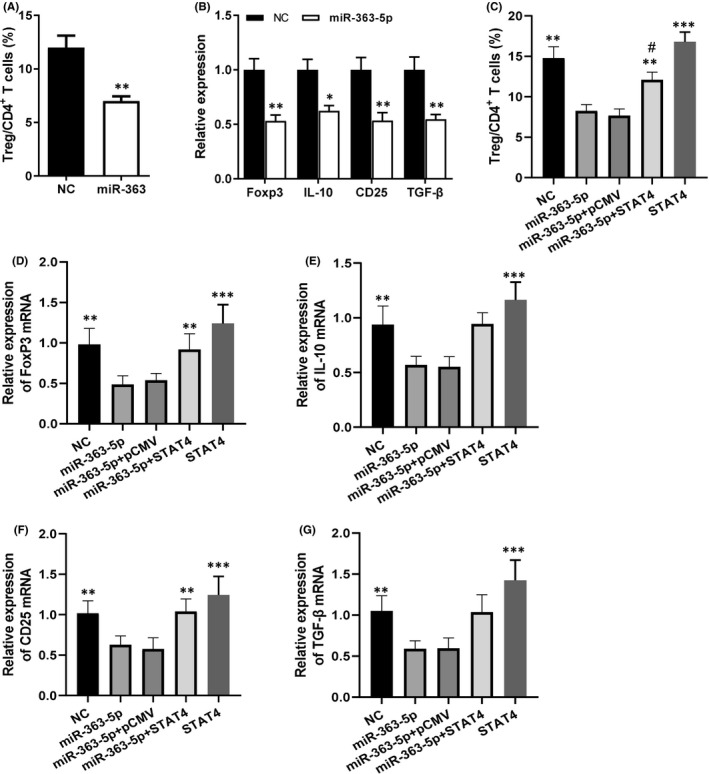
miR‐363‐5p suppressed differentiation and function of Treg cells via STAT4‐HSPB1‐Notch1 axis. (A, B) CD4+ T cells purified from PBMC of healthy individuals were transfected with miR‐363‐5p or its negative control sequence (NC) and then were stimulated and induced Treg cell differentiation. Five days later, the percentage of Tregs in CD4+ T cells was detected by flow cytometry (A) and the relative expression levels of Foxp3, IL‐10, CD25, TGF‐β mRNA were analysed by RT‐PCR (B). *: *p* < 0.05 vs. corresponding NC. **: *p* < 0.01 vs. corresponding NC. (C‐G) CD4+ T cells isolated from PBMC of healthy individuals were transfected, respectively, with miR‐363‐5p, negative control sequence (NC), miR363‐5 and pCMV empty vector (miR363‐5p + pCMV), miR363‐5 and STAT4 expression vector (miR363‐5p + STAT4), or STAT4 expression vector (STAT4), and then were stimulated and induced proliferation and differentiation of Treg cells. Five days later, the percentage of Tregs in CD4+ T cells was detected by flow cytometry (C), and the relative expression levels of Foxp3 (D), IL‐10 (E), CD25 (F) and TGF‐β (G) mRNA were analysed by RT‐PCR. **: *p* < 0.01 vs. the groups of miR‐363‐5p and miR363‐5p + pCMV; ***: *p* < 0.001 vs. the groups of miR‐363‐5p and miR363‐5p + pCMV; #: *p* < 0.05 vs. the group of STAT4. Data were presented as mean ± SEM. *t* test was used to determine the statistical significance in (A and B). One‐way anova was used in (C‐G)

**FIGURE 6:**
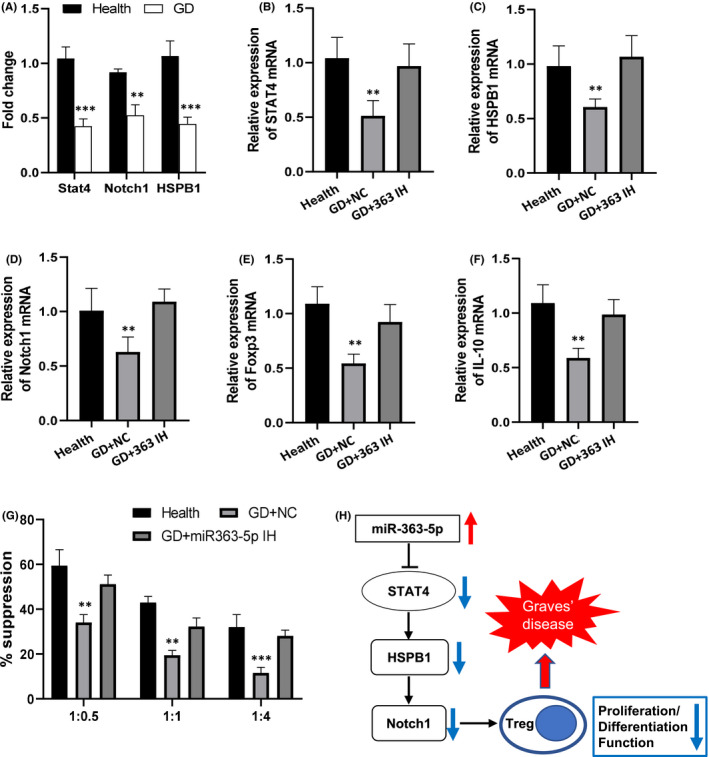
MiR‐363‐5p inhibition significantly improved Treg cell dysfunction in GD patients. (A) Fold changes in mRNA expression levels of STAT4, Notch1 and HSPB1 in Treg cells of peripheral blood of GD patients (GD) compared with those of healthy individuals (Health). **: *p* < 0.01 vs. corresponding health groups. ***: *p* < 0.001 vs. corresponding health groups. (B‐F) Peripheral Treg cells from GD patients were transfected with miR‐363‐5p inhibitor (GD+363 IH) or its negative control sequence (GD + NC); then, the relative expression levels of STAT4 (the target gene of miR‐363‐5p, B), HSPB1 (C), Notch1 (D), Foxp3 (E) and IL‐10 (F) compared with control group were analysed by RT‐PCR. Tregs in the peripheral blood of healthy individuals were used as control group (Health). **: *p* < 0.01 vs. other groups. (G) Immunosuppressive function of Treg cells from healthy individuals (Health) and from GD patients transfected with miR‐363‐5p inhibitor (GD+miR‐363‐5p IH) or negative control sequence (GD + NC). Horizontal coordinate is the ratio of Treg to effector T cells. **: *p* < 0.01 vs. other two groups with the same ratio. (H) Diagram of the mechanism by which miR‐363‐5p regulated Treg cells through STAT4‐HSPB1‐Notch1 axis. Data were presented as mean ± SEM. *t* test was used to determine the statistical significance in (A). One‐way anova was used in (B‐G)

To reveal the significance of the regulation of STAT4‐HSPB1‐Notch1 axis by miR‐363‐5p in Treg dysfunction of GD patients, CD4+T cells were purified from PBMC of healthy individuals and were transfected by miR‐363 agomir and negative control (NC) sequence. The results showed that miR‐363‐5p significantly reduced the proportion of Tregs after CD4+ T‐cell expansion compared with the control group transfected with NC sequence, indicated that miR‐363‐5p could significantly suppressed the proliferation/differentiation of Tregs (Figure 5A). Moreover, miR‐363‐5p also significantly inhibited the expression of Foxp3, IL‐10, CD25 and TGF‐β, which were closely related to Treg function (Figure 5B). The transfection of STAT4 expression vector significantly attenuated the inhibitory effects of miR‐363‐5p on proliferation/differentiation of Treg cells and the expression of Foxp3, IL‐10, CD25 and TGF‐β (Figure 5C‐G). Moreover, HSPB1 expression vector has similar effects to STAT4 expression vector (Figure S3C‐G). In addition to the upregulation of miR‐363‐5p, the expression of STAT4, HSPB1 and Notch1 was also significantly decreased in Tregs of GD patients compared with healthy subjects (Figure 6A). Moreover, miR‐363‐5p inhibitor significantly upregulated the expression of STAT4 and its downstream molecules HSPB1 and Notch1 as well as Foxp3 and IL‐10 in Tregs (Figure 6B‐F), thereby significantly improved the immunosuppressive function of Tregs in GD patients (Figure 6G). All these results proved that miR‐363‐5p could inhibit the differentiation/proliferation and function of Treg through STAT4‐HSPB1‐Notch1 axis and therefore might play important roles in the immune abnormalities of GD patients.

## 4 DISCUSSION

Treg cells are critical regulators of immune tolerance and inflammation, and its number and/or function are significantly reduced in various autoimmune diseases such as systemic lupus erythematosus and psoriasis.^4^, ^5^ Our findings showed that the proportion and function of Tregs in the peripheral blood of GD patients were significantly decreased compared with healthy individuals, which was concordance with previous studies.^7^, ^9^ However, some studies suggested that the number of peripheral Treg cells did not significantly change in GD patients, but rather that their function is impaired. Meanwhile, other studies showed that there were no obvious changes in the number and function of Tregs, and the changes in other cells or the microenvironment actually caused the Treg dysfunction.^6^ The possible reasons for these discrepancies are very complicated and may lie in the differences in races of GD patients, treatments, stages of disease and the assay methods and/or used marker molecules for Tregs.

Many studies have been conducted on the function of miR‐363‐3p. However, most of them focused on cancer and found that miR‐363‐3p was mainly involved in the development of solid tumours.^24^ MiR‐363‐3p was also reported to be associated with the differentiation and function of Th17 cells and played an important role in immune regulation.^25^ Nevertheless, there are only a few studies on miR‐363‐5p that showed its roles in the function of vascular endothelial cells and the development of tumours.^26^, ^27^ The present study demonstrated for the first time that miR‐363‐5p also participated in the regulation of proliferation/differentiation and function of Treg cells and had important immunomodulatory roles.

STAT4 is an important transcription factor and the essential regulator of the development and function of immune system. STAT4 can regulate the differentiation and development of Th1 and Th2 cells.^28^ STAT4 has been reported to suppress Treg development and to play key roles in the inhibitory effect of IL‐12 on Tregs.^29^ Foxp3 expression was upregulated, and the number of Tregs was increased in Stat4/Stat6/T‐bet triple‐knockout (TKO) mice.^30^ However, Stat4 knockout had no significant effect on nTreg cells.^29^ Studies also showed that STAT4 played active roles in the development and function of Treg. STAT4 knockout aggravated the immune abnormalities and hyperthyroidism of GD mice and accelerated the progress of GD,^31^, ^32^ which is consistent with our findings and indicated that STAT4 could promote the function and differentiation of Treg cells. One reason for such contradictory results is probably due to the difference in the species from which the cells are derived; namely, Tregs were derived from mice in previous studies showing that STAT4 inhibited Treg cell function, while the CEM cells and Tregs used in our study were derived from humans. Another reason may be the difference in the disease models used in different studies. The conclusion that STAT4 inhibited Tregs was drawn from allergic lung inflammation and autoimmune colitis models,^29^, ^30^ while the study performed in GD model showed that STAT4 could protect and even promote Treg function,^31^ which was consistent with our findings in GD patients. This also suggests that the same gene may play different or even opposite roles in different diseases, which has been widely reported.^33^, ^34^, ^35^, ^36^ All these indicated that the roles of STAT4 in immune regulation including Tregs development, proliferation, differentiation and function were complicated and needed to be further investigated.

Notch1 is the key molecule in the regulation of T cells, and its role in autoimmune diseases remains obscure. Especially, there are still many contradictions in its role in the regulation of proliferation, differentiation and function of Treg cells.^37^ Several studies showed that blocking Notch1 signalling significantly inhibited the function and proliferation of Treg cells,^38^ which was consistent with our findings. However, some other studies showed that Notch1 pathway activation promoted Tregs apoptosis and inhibited its function^39^ Blocking Notch1 could promote the function and proliferation of Tregs through increasing STAT5 phosphorylation.^40^ The reasons and mechanisms for these conflicts remain unclear and might be lie in the differences in disease, animal models, inflammatory environments and cytokines. Obviously, more research is needed to fully reveal the roles of Notch1 in Treg cell function.

Our study revealed that STAT4 regulated Notch1 expression through heat‐shock protein family B (small) member 1 (HSPB1). HSPB1 is a highly conserved protein and suppresses apoptosis caused by stress signals.^41^ HSPB1 is reported to be highly expressed in many cancers and participate in tumour cell proliferation.^41^ Some studies also showed that HSPB1 was involved in the inflammatory response and had immunomodulatory activity.^42^, ^43^ We revealed for the first time that HSPB1 could participate in the dysfunction of peripheral Tregs in GD patients via mediating the regulation of Notch1 signalling by STAT4. However, the roles of HSPB1 in immune modulation and more autoimmune diseases are still unknown and deserve further study. It should be noted that miR‐363‐5p inhibitor had no significant effect on the expression of its target genes or downstream molecules in CEM cells. This might be due to the fact that miR‐363‐5p expression was already at a low level in CEM cells and the effect of its inhibitor was not obvious. However, due to the high level of miR‐363‐5p expression in Tregs from GD patients, the effect of its inhibitor on its expression and target genes becomes apparent. This phenomenon has also been reported in many studies. For example, miR‐155 inhibitor could significantly reduce cell proliferation in HuT102 cells expressing high levels of miR‐155, while this effect was far less pronounced in HuT78 cells expressing low level of miR‐155.^44^

In summary, in the present study, we found for the first time that miR‐363‐5p was significantly upregulated in peripheral Tregs of GD patients and played important roles in Treg dysfunction of GD patients; namely, miR‐363‐5p could suppress proliferation/differentiation and function of Tregs through STAT4‐HSPB1‐Notch1 axis, which might be an important mechanism for the decreased number and dysfunctions of Tregs in GD patients (Figure 6H). Our findings improved our knowledge of miRNA function and Tregs regulation and deepened our understanding of the mechanism of immune abnormalities in GD patients.

## CONFLICT OF INTERESTS

Authors declare no competing interests.

## AUTHOR CONTRIBUTIONS

**Xianlun Yin** involved in conceptualization (equal); data curation (equal); and writing—original draft (lead). **Junfeng Ge** contributed to formal analysis (equal) and investigation (equal). **Xiurong Ge** contributed to investigation (equal) and resources (equal). **Jing Gao** contributed to methodology (supporting) and validation (supporting). **Xinhuan Su** contributed to data curation (equal) and methodology (supporting). **Xiao‐Wei Wang** contributed to methodology (supporting) and validation (supporting). **Qunye Zhang** contributed to conceptualization (lead); supervision (equal); writing—original draft (supporting); and writing—review and editing (lead). **Zhe Wang** contributed to conceptualization (lead); funding acquisition (lead); project administration (equal); supervision (equal); and writing—review and editing (lead).

## Supporting information

Appendix S1

## Data Availability

The data that support the findings of this study are available from the corresponding author upon reasonable request.
