# Supplementary material for: MiR‐363‐5p modulates regulatory T cells through STAT4‐HSPB1‐Notch1 axis and is associated with the immunological abnormality in Graves' disease
Source: J Cell Mol Med. 2021 Aug 25;25(19):9364–77. doi: 10.1111/jcmm.16876 (PMC8500983; doi:10.1111/jcmm.16876)
Supplement: Supplementary file 1 — Appendix S1 [file JCMM-25-9364-s001.docx]

**Supplementary materials**

**Table S1: PCR Primers used in the present study**

| Gene | Primers | Primer sequence (5′ 3′) |
| --- | --- | --- |
| *Notch1* | forward | 5'- TGGACGACAACCAGAATGAG-3' |
|  | reverse | 5'- CATCCAGGTGCTGCTGAGT-3' |
| *STAT4* | forward | 5'-CACCTGCCACATTGAGTCAACTA-3' |
|  | reverse | 5'-TAAGACCACGACCAACGTACGA-3' |
| *HSPB1* | forward | 5'-GCGTGTCCCTGGATGTCAAC-3' |
|  | reverse | 5'- TGTATTTCCGCGTGAAGCAC-3' |
| *TGF-β* | forward | 5'- GCCCTGGACACCAACTATTG -3' |
|  | reverse | 5'- CGGTAGTGAACCCGTTGATGT -3' |
| *IL-10* | forward | 5'- TGCCTTCAGCAGAGTGAAGA-3' |
|  | reverse | 5'- GGGTCTTGGTTCTCAGCTTG-3' |
| *CD25* | forward | 5'- TTCATACCTGCTGATGTGGGG-3' |
|  | reverse | 5'- TTGGTTGTCCCAGGACGAGT-3' |
| *Foxp3* | forward | 5'- TCCCAGAGTTCCTCCACAAC-3' |
|  | reverse | 5'- ATTGAGTGTCCGCTGCTTCT-3' |
| *ACTIN* | forward | 5'- GGACTTCGAGCAAGAGATGG-3' |
|  | reverse | 5'-AGGAAGGAAGGCTGGAAGAG-3' |

**Supplementary figure legends**


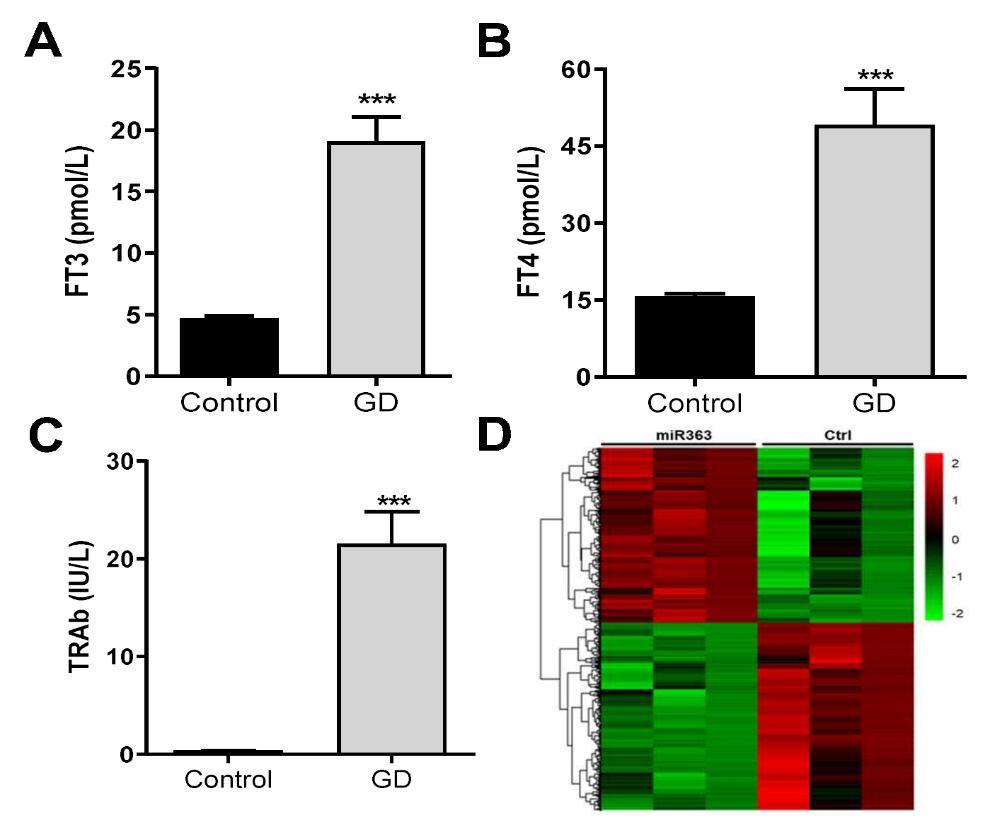


**Figure S1.** **(A-C)** The serum levels of FT3 (A), FT4 (B) and TRAb (C) in healthy individuals (Control) and GD patients (GD). **(D)** Heatmap of the fold changes of all genes with significantly changed expressions in CEM cells transfected with miR-363-5p mimic (miR363) compared to CEM cells transfected with negative control sequence (Ctrl). The Wald test with Benjamini-Hochberg correction for multiple tests was used for differential expression analysis. Fold change ≥1.5 or ≤0.67 and corrected p ≤0.05 were considered significant changes. The color bar represented log1.5(fold change). Data were presented as mean ± SEM. The *t*-test was used to detect significant changes. ***: *p* <0.001.


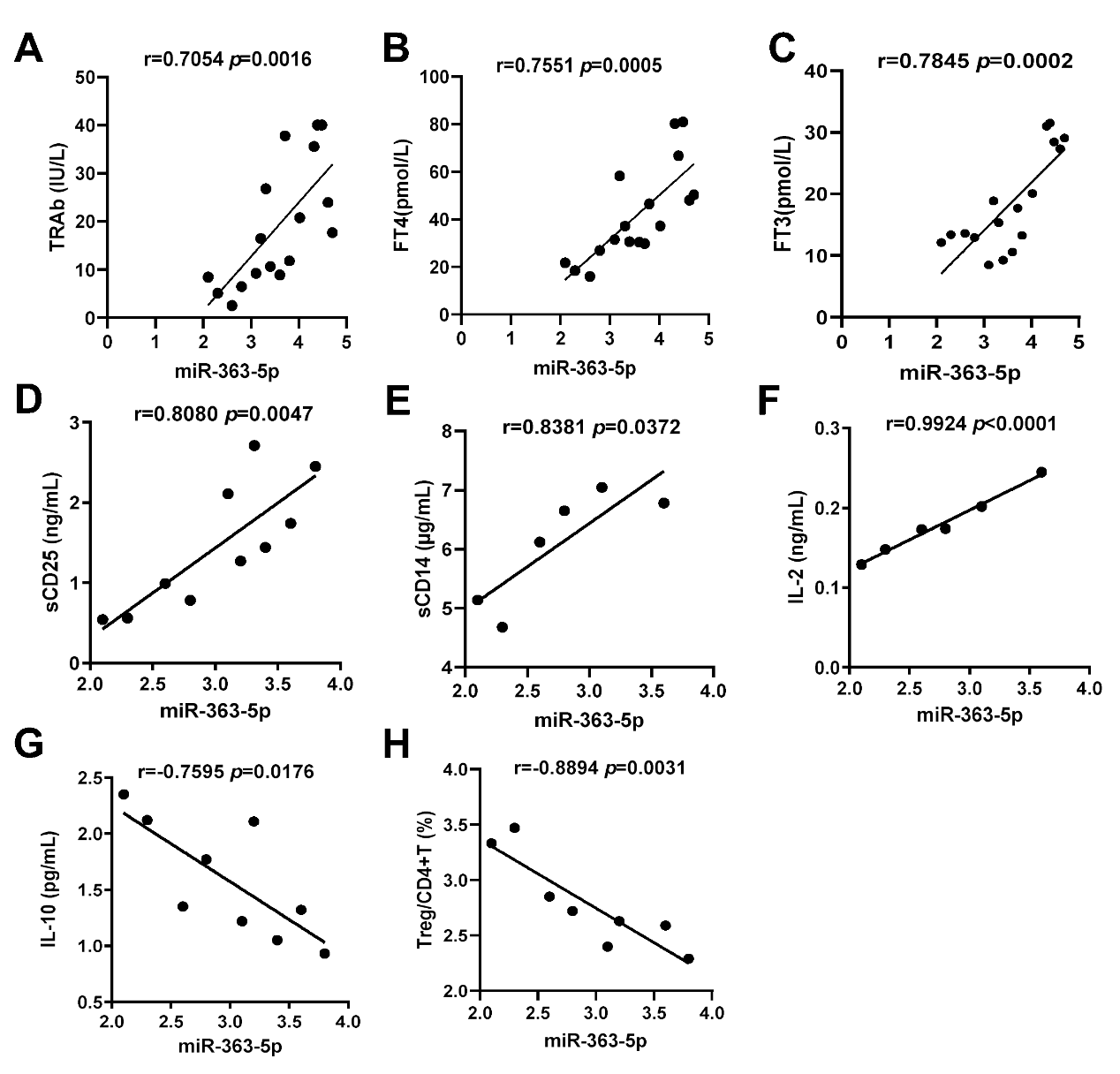


**Figure S2.** Linear regression between miR-363-5p expression level in peripheral blood Treg cells and the serum levels of TRAb **(A)**, FT4 **(B)** and FT3 **(C)**, sCD25 **(D)**, sCD14 **(E)**, IL-2 **(F)**, IL-10 **(G)** and the percentage of Treg cells in CD4+T cells **(H)**.


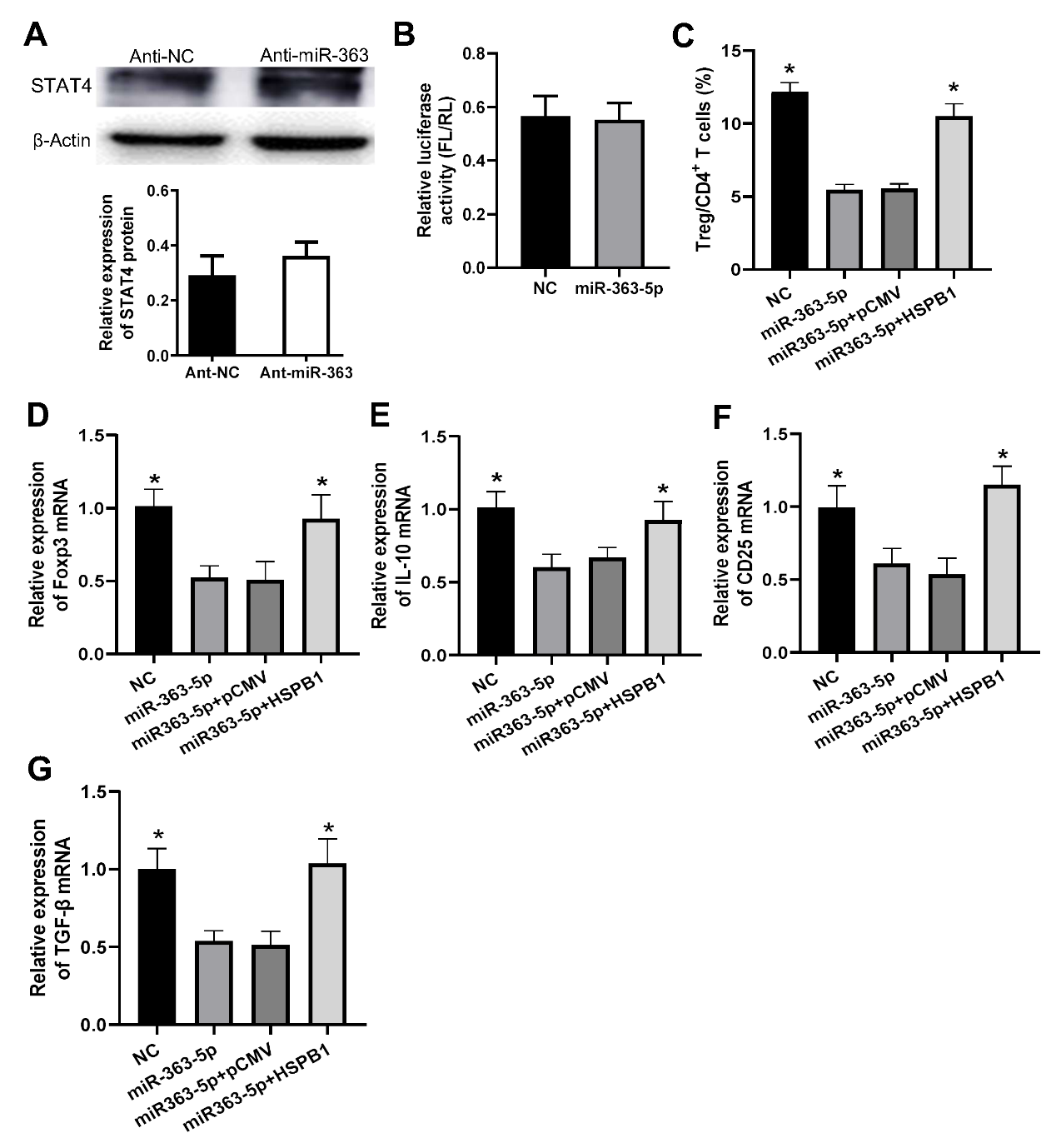


**Figure S3. (A)** Expression levels of STAT4 protein in CEM cells transfected with miR-363-5p inhibitor (anti-miR-363) or its negative control sequence (anti-NC). **(B)** The dual luciferase reporter vector containing HSPB1 3’UTR sequence was co-transfected with miR-363-5p or its negative control sequence (NC) in 293T cells. Then, the relative activity of luciferase was detected. Relative Luciferase active: fluorescence intensity ratios of firefly to sea cucumber normalized by those of 293T cells transfected with only reporter vector. **(C-G)** CD4+T cells purified from PBMC of healthy individuals were transfected with miR-363-5p, its negative control sequence (NC), miR363-5 and pCMV empty vector (miR363-5p+pCMV), or miR363-5 and HSPB1 expression vector (miR-363-5p+ HSPB1), and then were stimulated to induce Treg cell differentiation. Five days later, the percentage of Tregs in CD4+ T cells was detected by flow cytometry (C) and the relative expression levels of Foxp3 (D), IL-10 (E), CD25 (F) and TGF-β (G) mRNA were analyzed by RT-PCR. *: *p* <0.05 vs. the groups of miR-363-5p and miR363-5p+pCMV. Data were presented as mean ± SEM. The *t*-test was used to detect significant changes in A and B. One way-ANOVA was used in C-G.
